# Role of the rapid shallow breathing index to predict the success of mechanical ventilator liberation in acute respiratory failure

**DOI:** 10.1186/cc13490

**Published:** 2014-03-17

**Authors:** C Chanpharm, R Bhurayanontachai

**Affiliations:** 1 Prince of Songkla University, Hat Yai, Songkhla, Thailand

## Introduction

The rapid shallow breathing index (RSBI) is considered a good parameter to predict mechanical ventilator liberation. We hypothesized that the RSBI provides no benefit when clinical readiness criteria are met.

## Methods

**Figure 1:**
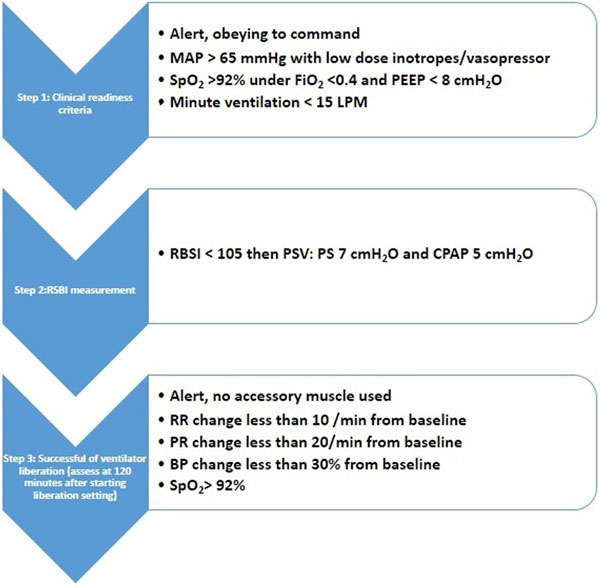
**Standard mechanical ventilator weaning protocol**.

Adults with acute respiratory who required MV for more than 24 hours, excluding COPD, were assessed daily as a liberation protocol (Figure 1). During the RSBI step, RSBI was recorded and blinded to the researcher. The liberation process was continued regardless of the RSBI result. The primary outcome was the success rate of mechanical ventilator liberation with or without RSBI.

## Results

Analysis of 120 cases with clinical characteristics as presented in Table 1. There was no statistically significant difference between using only clinical readiness and using clinical readiness and the RSBI (92% vs. 89%, *P *= 0.43).

**Table 1:** 

Character	CR	CR + RSBI	*P*value
Age	55.79 ± 19.6	56.62 ± 19.4	0.74
Male sex (%)	58.3	59.1	0.90
Success rate (%)	89.2	92.2	0.43

## Conclusion

The inclusion of RSBI in our standard mechanical ventilator liberation protocol for patients who met the clinical readiness criteria did not significantly increase the success rate of mechanical ventilator liberation.

